# ﻿A new species of *Svistella* Gorochov, 1987 from Xizang, China (Orthoptera, Trigonidiidae, Trigonidiinae)

**DOI:** 10.3897/zookeys.1193.117612

**Published:** 2024-03-06

**Authors:** Jing-Wen Hou, Yue Xu, Tian-Hao Hu, Zi-Heng Zhang, Shi-Yang Wu, Pu Gong, Zhu-Qing He

**Affiliations:** 1 School of Life Science, East China Normal University, Shanghai 200241, China East China Normal University Shanghai China; 2 Department of Environmental Science, Policy and Management, University of California, Berkeley, CA 94720, USA University of California Berkeley United States of America; 3 Zhejiang Provincial Center for Disease Control and Prevention, Hangzhou, Zhejiang, 310051, China Zhejiang Provincial Center for Disease Control and Prevention Hangzhou China

**Keywords:** *COI*, DNA barcoding, PCA, songs, taxonomy, Zayu

## Abstract

The genus *Svistella* Gorochov, 1987 includes 10 species from Asia, with nine documented in China. In this study, a new species, *Svistellayayun* He, **sp. nov.**, is described from Xizang, China. Morphologically, it resembles *S.rufonotata* (Chopard, 1932) but can be distinguished by a smaller inner tympanum, dark-brown setae on the 5^th^ segment of the maxillary palp, and a rounded apex on the ectoparamere. To validate our morphological inferences and support the description of *S.yayun***sp. nov.** as a new species, we performed a PCA based on bioacoustics parameters and molecular analysis. All *Svistella* species documented in China are distinguished by integrating their songs and DNA barcoding.

## ﻿Introduction

The genus *Svistella* Gorochov, 1987 belongs to the family Trigonidiidae Saussure, 1877, with all 10 species endemic to Asia (Cigliano et al. 2023). Over the past 50 years, the number of *Svistella* species has increased significantly. Initially, it comprised two species: *S.bifasciata* (= *Paratrigonidiumbifasciatum* Shiraki, 1911) and *S.rufonotata* (= *Anaxipharufonotata* Chopard, 1932), with the former designated as the type species. In 1993, *Anaxiphadubia* Liu & Yin, 1993 was described from Yunnan, China. Subsequently, He et al. (2009) reassigned *A.dubia* to the genus *Svistella* and described three new species: *S.tympanalis* He, Li & Liu, 2009, *S.anhuiensis* He, Li & Liu, 2009, and *S.fallax* He, Li & Liu, 2009. A new addition, *S.chekjawa* Tan & Robillard, 2012, was revealed in Singapore by Tan and Robillard (2012). Lu et al. (2018a) compared the morphological characters and DNA barcoding of the genera *Svistella* and *Paratrigonidium* Brunner von Wattenwyl, 1893 species, and described a new species, *S.fuscoterminata* He & Liu, 2018, from Yunnan, China. Additional contributions have included the description of *S.argentata* Ma, Jing & Zhang, 2019 by Ma et al. (2019), who also proposed *S.tympanalis* as a junior synonym of *S.rufonotata*. Li et al. (2021a) reported two additional species, *S.wuyong* He, 2021 and *S.malu* He, 2021, from Yunnan, China, based on morphological characteristics, calling-song analysis and molecular study (*COI*). Currently, nine *Svistella* species are known to occur in China (Cigliano et al. 2023).

The divergence of cricket songs usually precedes visible morphological differences, making song variations a significant driving factor for the divergence of cricket species (Otte 1992). Many male cricket species produce songs by stridulating their forewings to attract mates (Alexander 1962; Desutter-Grandcolas 1997; Mhatre and Balakrishnan 2006). Some perspectives indicated that songs may function as a significant mechanism for pre-mating isolation among species and a valuable tool in inferring species boundaries (Lu et al. 2018b; Tan et al. 2023). The song features are stable parameters within the same species (Fulton 1928), which may serve as cues for species recognition (Walker and Carlysle 1975). Thus, many new species are often initially identified based on their songs (Walker and Funk 2014). However, the calling songs can be easily influenced by temperature (Walker and Cade 2003; Jang and Gerhardt 2007). Song analysis is often combined with morphological observations and molecular analysis to identify new species (Chen et al. 2019; Tian et al. 2019; Li et al. 2021b).

During entomological surveys conducted in 2023, we first noticed unique songs different from any known cricket species. Our morphological, bioacoustics, and molecular analyses placed those newly collected individuals within the genus *Svistella*. However, the new specimens are different from any known *Svistella* species. Here we describe a new species, *Svistellayayun* He, sp. nov. from Xizang, China, and all Chinese *Svistella* species are characterized by a combination of their morphology, songs, and DNA barcoding.

## ﻿Materials and methods

### ﻿Sampling

We discovered this unknown species through its sounds during the night. Five individuals were collected from the wild and immediately preserved in 65% ethanol. After returning to the lab, a hind leg was preserved in anhydrous ethanol at −40 °C for molecular studies, and the remaining parts were preserved as dry specimens.

### ﻿Song recording and analyses

We recorded songs by using a SONY PCM A10 (ICX-0471) recorder. Three song recordings of *Svistellayayun* He, sp. nov. and all song recordings of other Chinese *Svistella* species from Li et al. (2021a) were replayed on a computer and analyzed using the Cool Edit software. *Svistellafallax* was not included in our PCA, because the audio file was lost, and the peak frequency was not available. Since most song recordings are shorter than 1 minute, we analyze the number of echemes in a randomly captured 10-second fragment from each recording, repeating this process 10 times. *Svistellaanhuiensis*, *S.bifasciata*, and *S.argentata* are regarded as continuous groups, and thus we define 10-second fragment of their song recordings as an echeme. We randomly select 10 echemes to analyze both their duration and the number of syllables in each. Bioacoustics characters—echeme interval, number of echemes per minute, number of syllables in each echeme, and peak frequency (Table 1)—are used in our PCA analysis. A principal component analysis (PCA) was performed in RStudio v. 2022.12.0 + 353 with *PCAtools* (Blighe and Lun 2023) based on collected bioacoustics parameters of the male calling songs (described below in song analysis).

**Table 1.** Features of *Svistella* spp. calling songs.

Species	Record site	Record time	Temperature (°C)	Echeme duration (s)	Echemes interval (s)	No. of echemes per minute	No. of syllables in each echeme	Peak frequency (Hz)	Data Source
*S.anhuiensis*	Wuyi Mountain, Fujian, China	20180911	27	10 +	0	—	—	7449.870 ± 83.908	This study
*S.argentata*	Jianfengling, Hainan, China	20090721	28	10 +	0	—	—	6245.520 ± 70.210	This study
*S.bifasciata*	Shenzhen, Guangdong, China	20190901	22	10 +	0	—	—	5496.450 ± 27.124	This study
*S.fallax*	Ankang, Shanxi, China	20190923	22	0.305 ± 0.010	0.320 ± 0.020	96.000 ± 0.000	16.400 ± 0.516	—	This study
*S.fuscoterminata*	Xishuangbanna, Yunnan, China	20171025	20	0.950 ± 0.020	2.024 ± 0.231	19.500 ± 2.550	29.700 ± 0.949	5127.970 ± 23.356	This study
*S.malu*	Kunming, Yunnan, China	20160926	25	0.440 ± 0.010	0.348 ± 0.030	83.400 ± 5.254	14.900 ± 0.994	6387.470 ± 23.299	This study
*S.rufonotata*	Wuyi Mountain, Fujian, China	20190312	18	0.150 ± 0.002	0.276 ± 0.004	138.000 ± 0.000	2.000 ± 0.000	5187.870 ± 30.050	This study
	Baisha, Hainan, China	20190403	18	0.150 ± 0.002	0.270 ± 0.004	138.000 ± 0.000	2.000 ± 0.000	4526.890 ± 29.970	This study
*S.wuyong*	Flowers-birds Market, China	20160920	24	0.443 ± 0.026	0.303 ± 0.049	87.600 ± 3.098	10.900 ± 0.738	5665.760 ± 72.207	This study
	Flowers-birds Market, China	20160930	24	0.393 ± 0.012	0.335 ± 0.030	82.800 ± 3.795	14.400 ± 0.516	6029.440 ± 44765	This study
	Flowers-birds Market, China	20160930	24	0.398 ± 0.017	0.407 ± 0.058	72.000 ± 7.483	14.200 ± 0.632	5930.810 ± 19.250	This study
	Flowers-birds Market, China	20160930	24	0.393 ± 0.017	0.461 ± 0.054	71.400 ± 3.406	13.700 ± 0.675	5942.970 ± 29.030	This study
	Flowers-birds Market, China	20160918	24	0.426 ± 0.027	0.273 ± 0.040	85.200 ± 2.530	11.300 ± 0.675	6242.520 ± 40.999	This study
*S.yayun*	Zayu, Xizang, China	20230709	20	0.939 ± 0.041	0.627 ± 0.045	42.000 ± 0.000	18.700 ± 0.949	5406.360 ± 116.973	This study
	Zayu, Xizang, China	20230709	20	0.992 ± 0.071	0.568 ± 0.037	37.200 ± 2.530	21.700 ± 1.494	5594.540 ± 34.098	This study
	Zayu, Xizang, China	20230709	20	0.967 ± 0.034	0.978 ± 0.135	31.800 ± 2.898	18.900 ± 0.738	5332.420 ± 65.809	This study

### ﻿Measurements

The sizes of the following body parts were measured on photographs by the ruler tool of Adobe Photoshop CC 2015.5. All the measurements are in millimeters (mm).

### ﻿Terminology

Terminology used to describe the male genitalia follows Tan and Robillard (2012).

Abbreviations:

**SZ** Body size (from head to tip of abdomen)

**FWL** Forewing length

**HFL** Length of hind femur

**PL** Pronotal length

**OL** Ovipositor length

**ec ap** Ectophallic apodeme

**en s** Endophallic sclerite

**ps ind** Pseudephiphallic indentation

**ps lo** Pseudephiphallic lophi

**r** Pseudephiphallic rami

**v** Ectophallic virgu (ectophallic fold)

All specimens are deposited in the Museum of Biology, East China Normal University (ECNU).

### ﻿DNA extraction and amplification

The total genomic DNA was extracted from the muscles of a hind leg by AxyPrep Genomic DNA Miniprep Kit (AXYGEN), according to the manufacturer’s instructions. The fragments of the mitochondrial cytochrome c oxidase subunit I gene (*COI*, 658 bp) were sequenced. Primers COBU (TYTCAACAAAYCAYAARGATATTGG) and COBL (TAAACTTCWGGRTGWCCAAARAATCA) were used (Pan et al. 2006). GenBank accession numbers are shown in Table 2.

**Table 2.** Collection information and *COI* GenBank accession number.

Genus	Species	Voucher	Collection site	GenBank	Data source
***Svistella***	*S.yayun*	4970	Zayu, Xizang, China	OR899297	This study
		4967	Zayu, Xizang, China	OR899298	This study
	*S.anhuiensis*	242	Chakou, Anhui, China	MG549837	Lu et al. 2018a
	*S.argentata*	302	Flowers-birds Market, China	MW647096	Li et al. 2021a
		333	Shenzhen, Guangdong, China	MW647097	Li et al. 2021a
	*S.bifasciata*	1254	Changjiang, Hainan, China	MW647098	Li et al. 2021a
		1427	Chebaling, Guangdong, China	MW647099	Li et al. 2021a
		260	Lishui, Zhejiang, China	MG549832	Lu et al. 2018a
		2014	Gutian Mountain, Zhejiang, China	MW647100	Li et al. 2021a
		33	Weng’ang, Guizhou, China	MG549831	Lu et al. 2018a
		2279	Tianmu Mountain, Zhejiang, China	MW647101	Li et al. 2021a
		318	Tianmu Mountain, Zhejiang, China	MW647102	Li et al. 2021a
		671	Hangzhou, Zhejiang, China	MG549833	Lu et al. 2018a
	*S.dubia*	637	Tengchong, Yunnan, China	MW647124	Li et al. 2021a
		740	Baoshan, Yunnan, China	MW647125	Li et al. 2021a
***Svistella***	*S.fallax*	1017	Tongjiang, Sichuan, China	MW647109	Li et al. 2021a
		2326	Xunyangba, Shaanxi, China	MW647110	Li et al. 2021a
		1513	Flowers-birds Market, China	MW647111	Li et al. 2021a
		1514	Flowers-birds Market, China	MW647112	Li et al. 2021a
	*S.fuscoterminata*	1133	Nabang, Yunnan, China	MW647113	Li et al. 2021a
		2274	Nabang, Yunnan, China	MW647114	Li et al. 2021a
		2307	Nabang, Yunnan, China	MW647115	Li et al. 2021a
		1161	Ruili, Yunnan, China	MW647116	Li et al. 2021a
		551	Xishuangbanna, Yunnan, China	MG549834	Lu et al. 2018a
		954	Xishuangbanna, Yunnan, China	MG549835	Lu et al. 2018a
	*S.malu*	1961	Tengchong, Yunnan, China	MW647103	Li et al. 2021a
		2288	Kunming, Yunnan, China	MW647104	Li et al. 2021a
		1960	Tengchong, Yunnan, China	MW647105	Li et al. 2021a
		289	Kunming, Yunnan, China	MW647106	Li et al. 2021a
		297	Kunming, Yunnan, China	MW647107	Li et al. 2021a
		2289	Kunming, Yunnan, China	MW647108	Li et al. 2021a
	*S.rufonotata*	1634	Baisha, Hainan, China	MW647117	Li et al. 2021a
		494	Mengla, Yunnan, China	MW647118	Li et al. 2021a
		1756	Wuyi Mountain, Fujian, China	MW647119	Li et al. 2021a
		243	Flowers-birds Market, China	MW647120	Li et al. 2021a
	*S.wuyong*	2318	Nabang, Yunnan, China	MW647121	Li et al. 2021a
		2320	Nabang, Yunnan, China	MW647122	Li et al. 2021a
		286	Flowers-birds Market, China	MW647123	Li et al. 2021a
***Amusurgus***	*A.genji*	317	Lin’an, Zhejiang, China	MT706087	He et al. 2020

### ﻿Molecular study

The *COI* sequences from newly reported species, along with 36 individuals and the outgroup *Amusurgusgenji* obtained from GenBank, were aligned using the MUSCLE method in MEGA 11 (Tamura et al. 2021). A distance tree was constructed employing the neighbor-joining (NJ) method following Kimura 2-parameter (K2P) model, with 0.19 gamma parameter and 95% partial deletion. The bootstrap test was performed with 10000 replicates. To define species partitions and estimate the number of molecular operational taxonomic units (MOTUs), we used two DNA-based species delimitation methods: Automatic Barcode Gap Discovery (ABGD, Puillandre et al. 2012) and Assemble Species by Automatic Partitioning (ASAP, Puillandre et al. 2021).

## ﻿Taxonomy

**Order Orthoptera**

**Family Trigonidiidae**

**Subfamily Trigonidiinae**

### ﻿Genus Svistella

Taxon classificationAnimaliaOrthopteraTrigonidiidae

Gorochov, 1987

02AE3805-3279-57E3-BA8D-705C88840300

Svistella Gorochov, 1987; He et al. 2009; Tan and Robillard 2012; Lu et al. 2018; Ma et al. 2019; Li et al. 2021.

#### Type species.

*Svistellabifasciata* (= *Paratrigonidiumbifasciatum* Shiraki, 1911).

### ﻿ Svistella yayun

Taxon classificationAnimaliaOrthopteraTrigonidiidae

He sp. nov.

4F6B19D8-2A7A-5BC0-B11B-401A00099841

https://zoobank.org/BD70EE0F-2270-44F8-8751-401E8DE434D3

**Figure 1.**
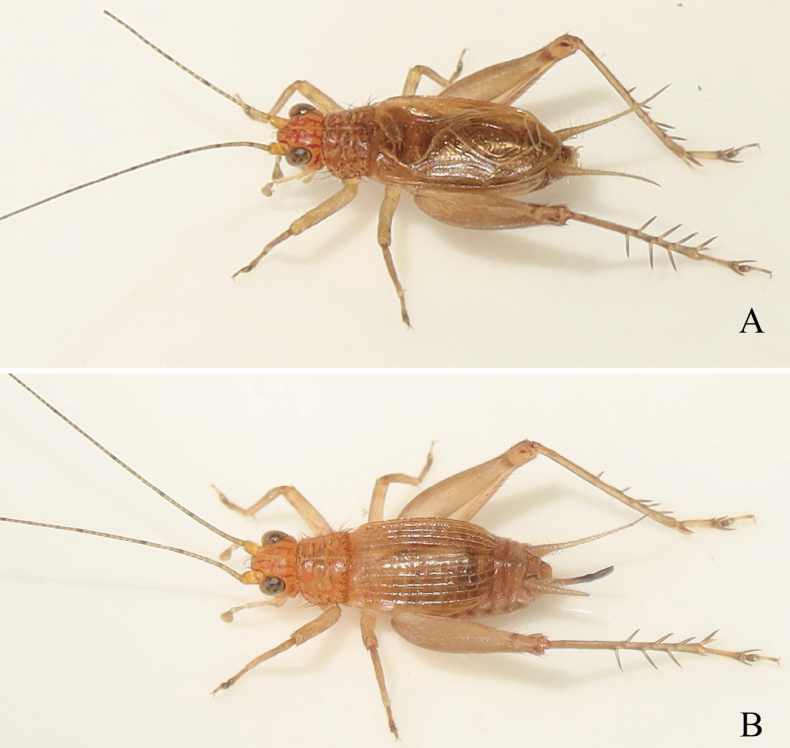
Living *Svistellayayun* He, sp. nov. **A** male **B** female.

**Figure 2.**
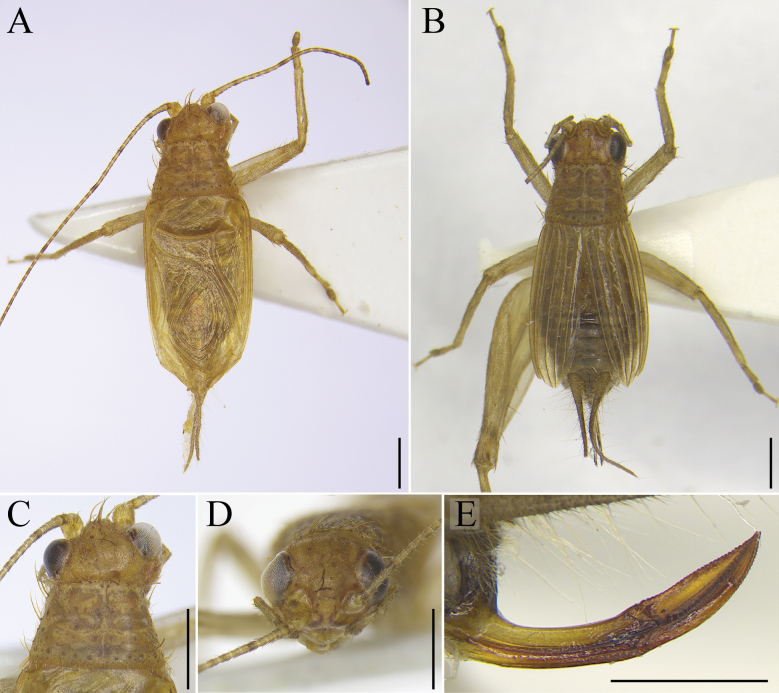
*Svistellayayun* He, sp. nov., male holotype, ECNU 4969, and female paratype, ECNU 4968 **A** habitus of male **B** habitus of female **C** male head and pronotum in dorsal view **D** male face in front view **E** female ovipositor in lateral view. Scale bars: 1 mm.

**Figure 3.**
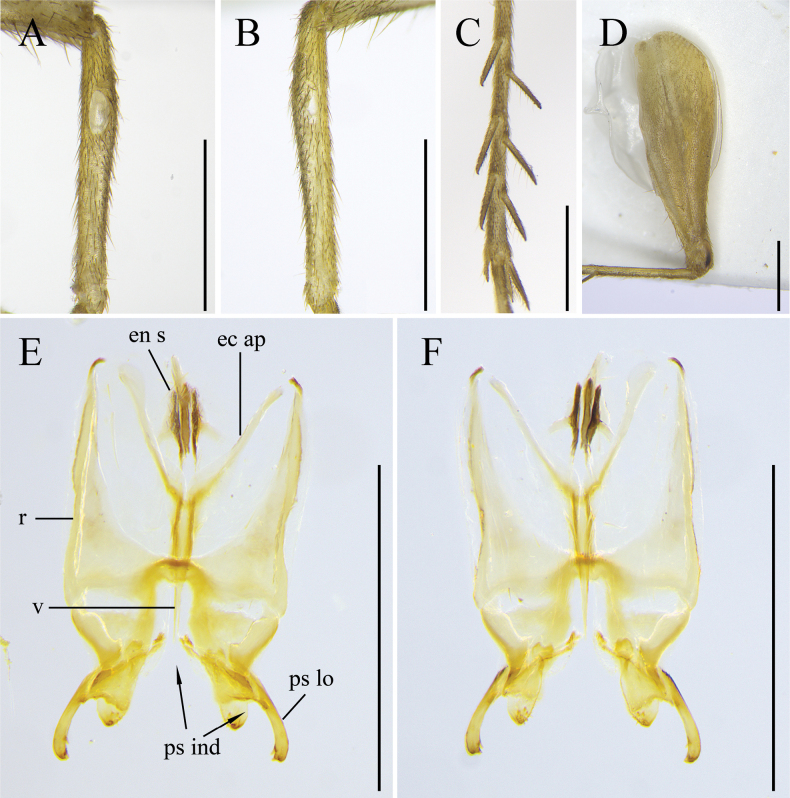
*Svistellayayun* He, sp. nov. **A** outer side of fore tibiae **B** inner side of fore tibia **C** hind tibiae in dorsal view **D** hind femora in lateral view **E** male genitalia in dorsal view **F** male genitalia in ventral view. All images are from holotype, ECNU 4969. Scale bars: 1 mm.

**Figure 4.**
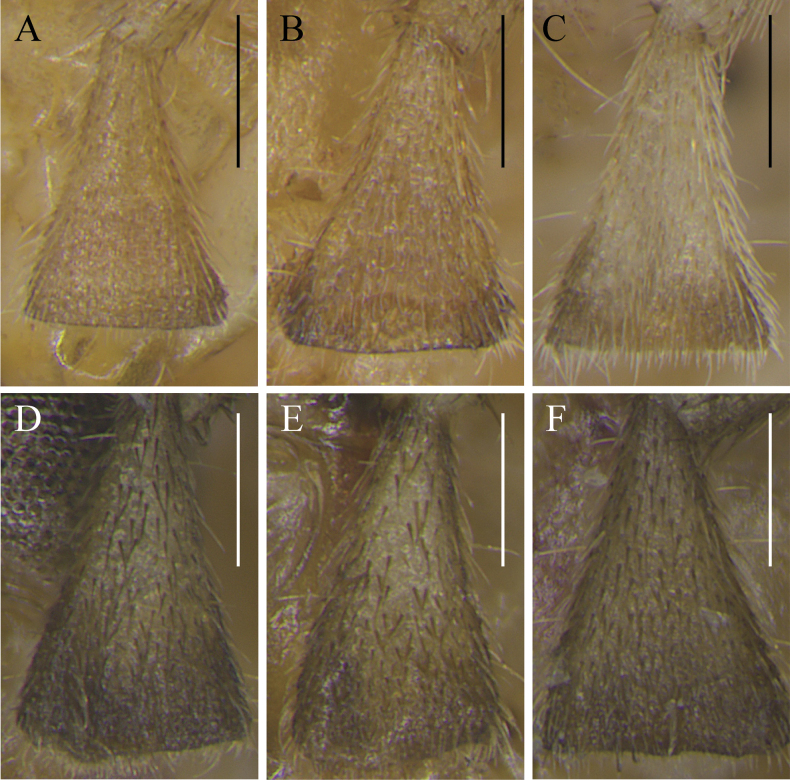
The 5^th^ segment of left maxillary palp of *Svistellarufonotata* (**A–C**) and *S.yayun* He, sp. nov. (**D, E**) images **A–C** from specimens ECNU 494, ECNU 1634, and ECNU 1756, respectively **D–F** from paratypes ECNU 4961, ECNU 4967, and holotype ECNU 4969, respectively. Scale bars: 250 μm.

Figs 1A, B , 2A–E , 3A–F , 4D–F

#### Diagnosis.

**Figure 5.**
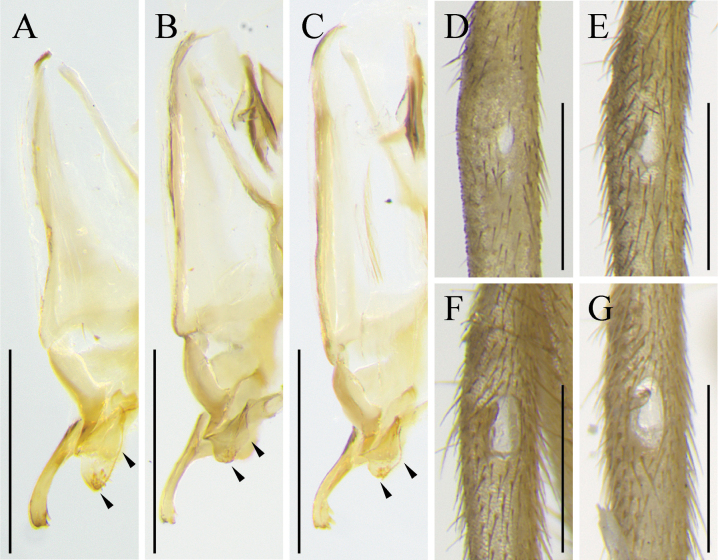
Male genitalia and right inner tympanum of *Svistellarufonotata* and *S.yayun* He, sp. nov. **A** male genitalia of *S.yayun* He, sp. nov. in ventral view, holotype, ECNU 4969 **B, C** male genitalia of *S.rufonotata* in ventral view from specimens ECNU 266 and ECNU 494, respectively **D, E** inner tympanum of *S.yayun* He, sp. nov. from a paratype ECNU 4961 and the holotype, ECNU 4969, respectively **F, G** inner tympanum of *S.rufonotata* from specimens ECNU 494 and ECNU 1756, respectively. Black arrows indicate the tip and a lateral process of ectoparamere. Scale bars: 0.5 mm.

The new species is characterized as follows: small to medium body size for the genus; dark-brown setae on the 5^th^ segment of maxillary palp; small, inner tympanum; hind femora without black stripe; tegmina unicolor. It is morphologically similar to *S.rufonotata* but differs in having dark-brown setae on the 5^th^ segment of maxillary palp (Fig. 4A–F), an ectoparamere with rounded corner (Fig. 5A–C), and a smaller inner tympanum (Fig. 5D–G).

#### Materials examined.

***Holotype***: China • ♂; Xizang, Zayu; 28°28.20'N, 97°01.22'E; 1565 m); 9 July 2023; He Zhu-Qing leg.; ECNU 4969. ***Paratypes***: 2♂, ECNU 4961, ECNU 4970 & 2♀, ECNU 4967, ECNU 4968; same data as for holotype.

#### Description.

**Male.** Body size small. Head slightly wider than anterior margin of pronotum, occiput slightly convex (Figs 1A, 2A); frontal rostrum about as wide as first antennal segment, with two rows of setae extending to vertex (Fig. 2C); vertex not dorsally flattened; antennae long and pubescent; compound eyes slightly protruding forwards; 5^th^ segment of maxillary palpi triangular and swollen (Fig. 2D). Pronotum with setae, posterior margin widened; fore tibiae armed with two oval tympanums, with outer one bigger than inner one (Fig. 3A, B); hind tibiae bearing three pairs of dorsal spurs (Fig. 3C) and five apical spurs (two internal ones distinctly longer and three external ones shorter); tegmina barely reaching apex of abdomen. Cercus with long, thin hair.

***Genitalia*.** Pseudepiphallus separated into two lateral parts joined by a straight sclerotized bridge. Pseudepiphallic lophi curved inwards with 3 or 4 forks apically. Posterior marginal area of endoparameron with minute teeth and short setae (Fig. 3E, F).

**Female.** Similar to male (Fig. 1B). Tegmina slightly convex and not extending to abdominal apex (Fig. 2B). Ovipositor long and curved upwards, finely denticulate on dorsal and ventral sides (Fig. 2E).

***Coloration*.** Body brown; legs yellowish brown. Head orange and marked with five vertical red stripes extending to pronotum in dorsal view. Setae on the 5^th^ segment of maxillary palpi dark brown (Fig. 4D, E). Abdomen with 1 lateral red spot on both sides of each abdominal segment in female. Each hind femur with a dark-brown stripe near knees when alive (Fig. 1A, B) but disappearing after drying (Fig. 3D). Ovipositor dark brown; apical half darker than basal part.

***Variation*.** A paratype (ECNU 4961) has seven dorsal spurs on the hind tibiae (four internal ones and three external ones), while all the other examined specimens bear six dorsal spurs on the hind tibiae.

#### Measurements.

***Holotype***: ♂ BL 5.63, PL 1.09, FWL 4.08, HFL 4.35; ***Paratypes***: ♂ BL 5.86–6.16, PL 1.05–1.18, FWL 4.09–4.14, HFL 4.07–4.30; ♀ BL 5.55–5.63, PL 1.19–1.27, FWL 3.36–3.41, HFL 4.19–4.30, OL 2.05–2.07.

#### Distribution.

China (Xizang).

#### Etymology.

The specific epithet *yayun* is for the Chinese phonetic alphabet, 雅韵, which means “beautiful music”.

##### ﻿Molecular study

**Figure 6.**
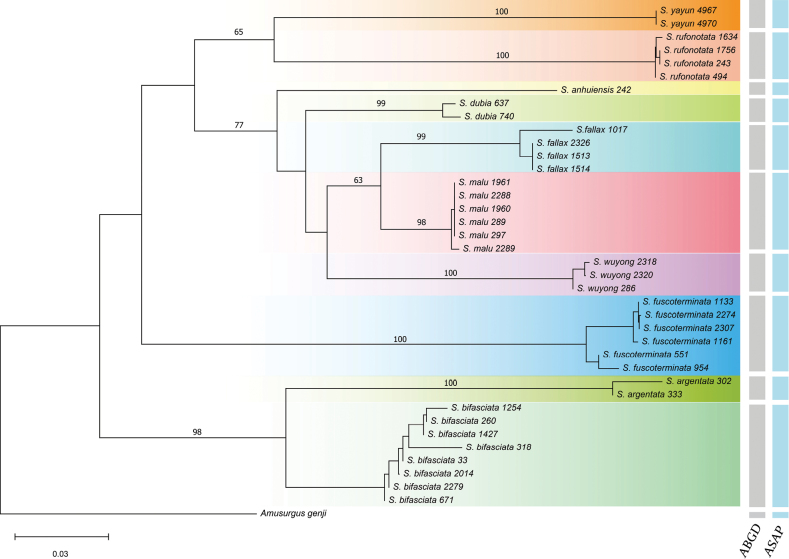
Distance tree of *Svistella* species based on *COI* genes. Rooted by *Amusurgusgenji*, the tree was constructed using the neighbor-joining (NJ) method with the Kimura 2-parameter model and a 0.19 gamma parameter. Topology supports of major nodes are indicated above branches by bootstrap value. Two DNA barcode-based (ABGD, ASAP) delimitation methods are represented by vertical bars in grey and blue, respectively, on the right side of the tree.

In total, 39 *COI* sequences including our newly described *S.yayun* He, sp. nov., as well as the *COI* sequence of *Amusurgusgenji* as the outgroup, were obtained. The results of the two molecular methods identified 11 putative species, which largely conform to the distance tree inferred from the NJ topology and are all consistent with separating *S.yayun* He, sp. nov. as a species (Fig. 6).

##### ﻿Song analysis

**Figure 7.**
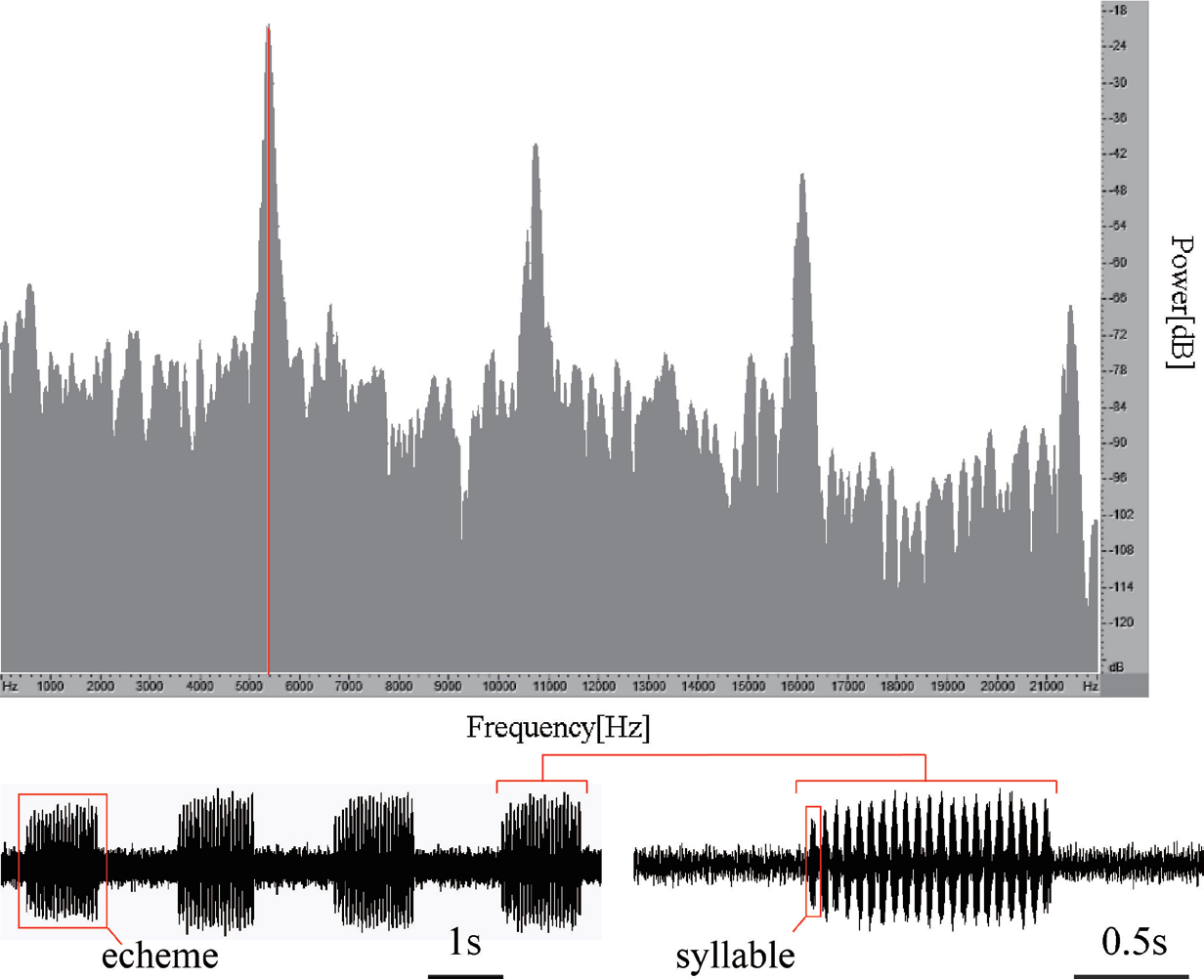
Spectrogram (upper) and oscillograms (lower) of male calling songs of *Svistellayayun* sp. nov. The red line in spectrogram indicates the peak frequency.

The calling song of *Svistellayayun* sp. nov. is stereotyped with 37.11 ± 5.00 [30–42] echemes/minute. Each echeme continues 0.966 ± 0.049 [0.901–1.094] second and consists of 19.77 ± 1.060 [18–24] syllables (Fig. 7). The peak frequency is 5332.420–5594.540 Hz (Fig. 7). Although *S.rufonotata* and *S.yayun* are similar morphologically, they can be distinguished by their songs. The characteristics of the songs of all included species in PCA are shown in Table 1. The species *S.fallax* was not included in PCA, because the audio file was lost, and the peak frequency for this species was unavailable.

**Figure 8.**
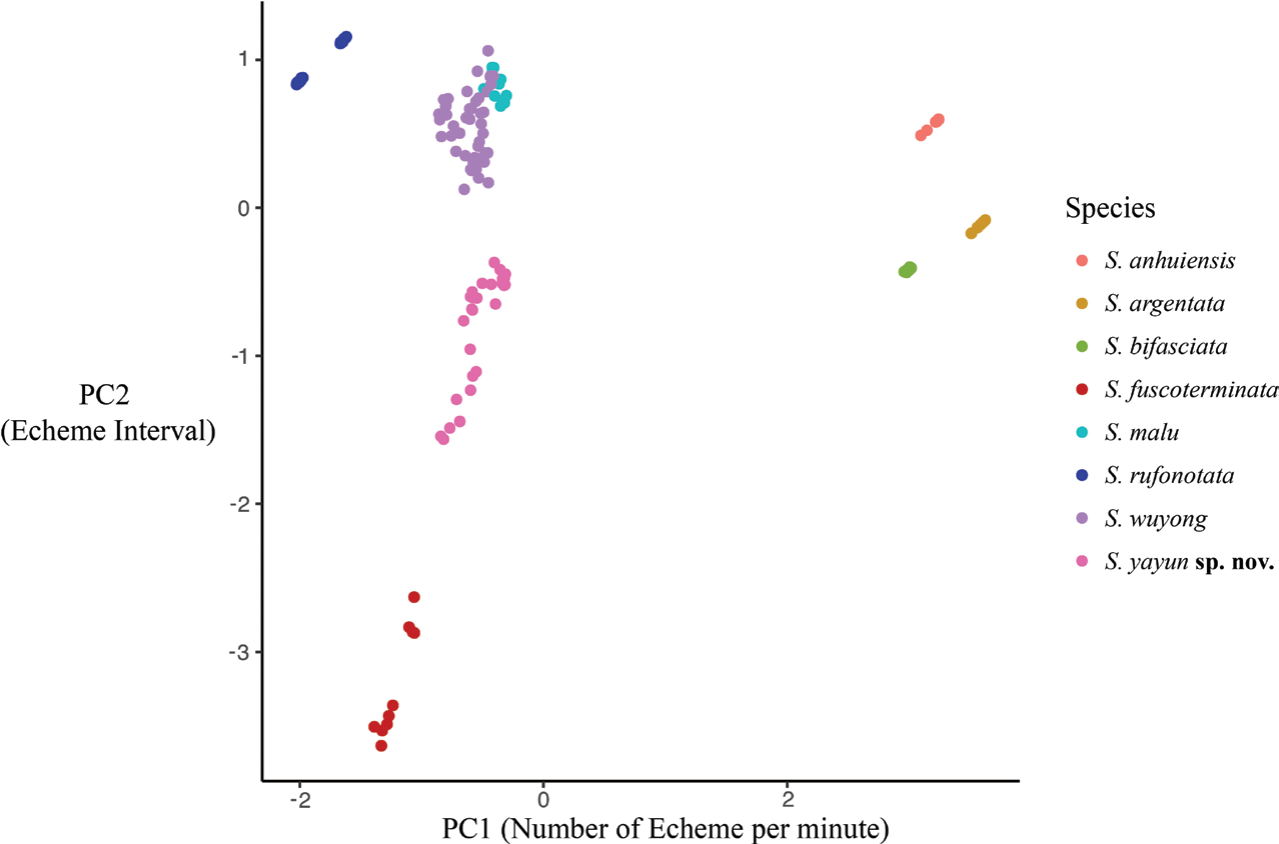
PCA figure description of *Svistella* species songs. Scatter plot of PC1 and PC2 of PCA based on bioacoustics measurements. Except for *S.wuyong* and *S.malu*, the remaining species can be clearly identified through the analysis of their calling songs.

PCA results are shown in Fig. 8. The extracted components PC1 eigenvectors accounted for 57.90% of the variance, PC2 for 24.04%, PC3 for 15.05%, PC4 for 2.02%, and PC5 for 0.99%. Except for *S.wuyong* and *S.malu*, the remaining species can be clearly identified via the analysis of their calling songs.

##### ﻿Other materials examined

*Svistellarufonotata*: China • ♂; bought from Flowers-birds Market; September 2016; He Zhu-Qing; ECNU 266 • ♂; Yunnan, Mengla, Wangtianshu (望天树景区); 21°37.04'N, 101°33.56'E; 26 April 2017; He Zhu-Qing leg.; ECNU 494 • ♂; Hainan, Baisha, Nankai; 19°04.78'N, 109°22.57'E; 18 March 2019; He Zhu-Qing leg.; ECNU 1634 • ♂; Fujian, Wuyishan; 27°41.28'N, 117°44.38'E; 22 September 2018; He Zhu-Qing leg.; ECNU 1756.

## ﻿Discussion

In this study, the distance tree based on *COI* sequences shows that *Svistellayayun* sp. nov. is separate and distinct from other *Svistella* species and the reconstructed tree topology aligns with the earlier study using the same gene for eight species (Li et al. 2021a). Morphologically, *S.yayun* sp. nov. is similar to *S.rufonotata*, but it can be distinguished by the dark-brown setae on the 5^th^ segment of maxillary palp (light colored in *S.rufonotata*, Fig. 4), smaller inner tympanum with only 83.3–119.1 μm in long diameter (185.7–214.3 μm in *S.rufonotata*, Fig. 5D–G) and rounded apex of the ectoparamere (relatively abrupt in *S.rufonotata*, Fig. 5A–C). Additionally, bioacoustics PCA unveils that the songs of *S.yayun* sp. nov. form a distinct cluster compared to *S.rufonotata* and all other previously described species. Collectively, our molecular, morphological, and bioacoustics analyses provide support for recognizing *S.yayun* sp. nov. as a new species.

Probably due to its small size, it is challenging to collect or observe *S.yayun* sp. nov. in the field without relying on its distinctive songs. In our prior experience, we have occasionally identified new orthopteran species in the field based on their unique songs (Liu et al. 2018; Chen et al. 2019; Tian et al. 2019; Li et al. 2021b). Despite the crucial role of song in the speciation and evolution of Orthoptera, bioacoustic data can significantly enhance our understanding of orthopteran taxonomy, particularly considering that the divergence in songs among species often precedes noticeable morphological differences (Otte 1992). This highlights the importance of incorporating bioacoustic data as a defining characteristic in studies of Orthoptera.

Discoveries of new *Svistella* and other new species of Trigonidiidae often reveal the influence of geographical barriers and communication signals on species isolation (Mendelson et al. 2004; Grace and Shaw 2011; Stamps and Shaw 2019; Li et al. 2021a). Most *Svistella* species have allopatric distributions, while parapatric *Svistella* species produce distinctive songs to attract females (Li et al. 2021a). This highlights the role of both geographical barriers and bioacoustic signals in isolating these species. Moreover, despite bioacoustic signals, chemical, and/or tactile cues may contribute to species’ recognition to isolate different *Svistella* species in contact zones (Mullen et al. 2007). Similar patterns have been observed in other trigonidiid species, such as *Laupala* (Grace and Shaw 2011; Stamps and Shaw 2019). In line with recent studies, the discovery of *S.yayun* sp. nov. may underscore the association of geographical barriers, behavioral ecology, and *Svistella* speciation.

## Supplementary Material

XML Treatment for Svistella

XML Treatment for Svistella yayun

## ﻿Additional information

### Author ORCIDs

Jing-Wen Hou https://orcid.org/0009-0006-9395-8040

Yue Xu https://orcid.org/0009-0005-7235-1613

Tian-Hao Hu https://orcid.org/0000-0003-3210-961X

Zi-Heng Zhang https://orcid.org/0009-0002-1944-2776

Shi-Yang Wu https://orcid.org/0000-0001-6812-1878

Pu Gong https://orcid.org/0000-0002-7050-6643

Zhu-Qing He https://orcid.org/0000-0003-4304-767X

### Data availability

All of the data that support the findings of this study are available in the main text or Supplementary Information.

## Supplementary materials

Supplementary material 1 A song recording of *Svistellayayun* 1 Data type wav https://binary.pensoft.net/file/997407This dataset is made available under the Open Database License (http://opendatacommons.org/licenses/odbl/1.0/). The Open Database License (ODbL) is a license agreement intended to allow users to freely share, modify, and use this Dataset while maintaining this same freedom for others, provided that the original source and author(s) are credited.Author: Zhu-Qing He

Supplementary material 2 A song recording of *Svistellayayun* 2 Data type wav https://binary.pensoft.net/file/997408This dataset is made available under the Open Database License (http://opendatacommons.org/licenses/odbl/1.0/). The Open Database License (ODbL) is a license agreement intended to allow users to freely share, modify, and use this Dataset while maintaining this same freedom for others, provided that the original source and author(s) are credited.Author: Zhu-Qing He

Supplementary material 3 A song recording of *Svistellayayun* 3 Data type wav https://binary.pensoft.net/file/997409This dataset is made available under the Open Database License (http://opendatacommons.org/licenses/odbl/1.0/). The Open Database License (ODbL) is a license agreement intended to allow users to freely share, modify, and use this Dataset while maintaining this same freedom for others, provided that the original source and author(s) are credited.Author: Zhu-Qing He

Supplementary material 4 A video of a chirping male *Svistellayayun* Data type mp4 https://binary.pensoft.net/file/997410This dataset is made available under the Open Database License (http://opendatacommons.org/licenses/odbl/1.0/). The Open Database License (ODbL) is a license agreement intended to allow users to freely share, modify, and use this Dataset while maintaining this same freedom for others, provided that the original source and author(s) are credited.Author: Zhu-Qing He
